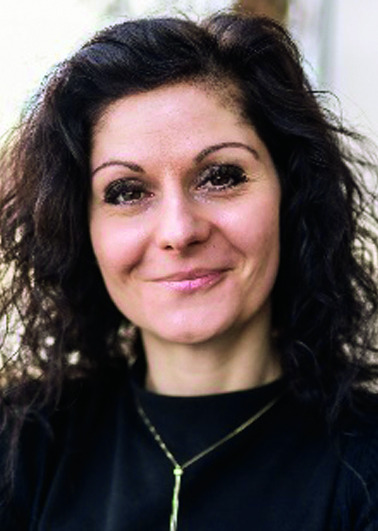# The Mysterious World of Non‐Canonical Caps – What We Know and Why We Need New Sequencing Techniques

**DOI:** 10.1002/cbic.202400604

**Published:** 2024-10-27

**Authors:** Flaminia Mancini, Hana Cahova

**Affiliations:** ^1^ Chemical Biology of Nucleic Acids Institute of Organic Chemistry and Biochemistry of the CAS Flemingovo náměstí 2 Prague 6 Czech Republic; ^2^ Charles University Faculty of Science Department of Cell Biology Vinicna 7 Prague 2 Czech Republic

**Keywords:** Mass spectrometry, RNA, RNA capping, RNA sequencing, RNA structures

## Abstract

It was long believed that viral and eukaryotic mRNA molecules are capped at their 5′ end solely by the N^7^‐methylguanosine cap, which regulates various aspects of the RNA life cycle, from its biogenesis to its decay. However, the recent discovery of a variety of non‐canonical RNA caps derived from metabolites and cofactors — such as NAD, FAD, CoA, UDP‐glucose, UDP−N‐acetylglucosamine, and dinucleoside polyphosphates — has expanded the known repertoire of RNA modifications. These non‐canonical caps are found across all domains of life and can impact multiple aspects of RNA metabolism, including stability, translation initiation, and cellular stress responses. The study of these modifications has been facilitated by sophisticated methodologies such as liquid chromatography‐mass spectrometry, which have unveiled their presence in both prokaryotic and eukaryotic organisms. The identification of these novel RNA caps highlights the need for advanced sequencing techniques to characterize the specific RNA types bearing these modifications and understand their roles in cellular processes. Unravelling the biological role of non‐canonical RNA caps will provide insights into their contributions to gene expression, cellular adaptation, and evolutionary diversity. This review emphasizes the importance of these technological advancements in uncovering the complete spectrum of RNA modifications and their implications for living systems.

## Graphical abstract

This review describes sophisticated methodologies such as LC–MS that enabled the identification of 5’ non‐canonical RNA caps and also surveys so far developed sequencing methods, evaluating their advantages and disadvantages. It emphasizes the need for developing new, reliable sequencing techniques to identify RNA types with non‐canonical caps enabling to study their biological roles.

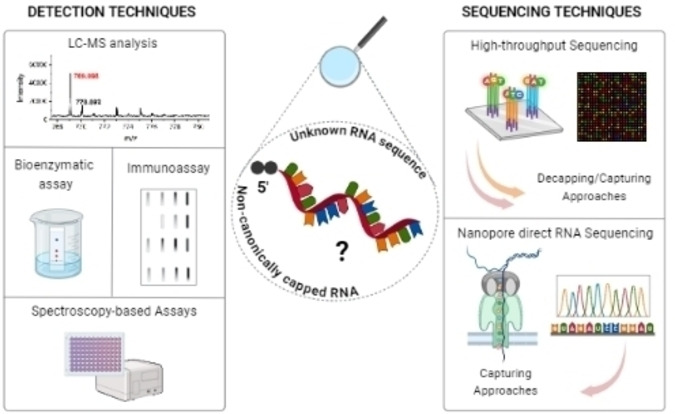

## 1 Introduction

The fundamental principle of molecular biology, formulated by its central dogma, outlines the transmission of genetic information from DNA to RNA and ultimately to proteins within a cell. However, Crick's traditional view is now undergoing a transformation, as the role of RNA expands far beyond a mere intermediary. Indeed, the intricate functionality of RNA is not solely determined by the information stored in its sequence. All aspects of gene expression have the potential to be regulated, providing evolution with various mechanisms to generate phenotypic diversity. Processes like splicing, particularly alternative splicing, stand as potent diversifying pathways for RNAs. Moreover, the potential for chemical modifications and RNA editing at each nucleoside further enriches this multilayered regulatory landscape. Chemical RNA modifications ubiquitously exist across all three kingdoms of life.^[1]^ Presently, more than 170 RNA modifications have been identified in prokaryotes, archaea and eukaryotes.^[1]^ These modifications exist in the majority of cellular RNAs, including messenger RNA (mRNA), transfer RNA (tRNA), ribosomal RNA (rRNA) and small non‐coding RNA (sncRNA). Modification events can occur internally along the RNA strand, involving N6‐methyladenosine (m⁶A),^[2]^ 5‐methylcytidine (m⁵C),[^3^, ^4^] ribose methylation (2’‐O−Me),^[5]^ pseudouridylation (ψ)^[6]^ and others. Modifications can also occur at the RNA termini, such as the addition of a 5′ cap, termed “5′ capping”, or the attachment of a polyadenylated or polynucleotide tail at the 3′ end. These terminal modifications play pivotal roles in RNA stability, translation initiation and mRNA export from the nucleus, collectively shaping the destiny of the RNA molecule. In eukaryotes, a thoroughly documented and evolutionarily conserved 5′ modification process involves a series of three enzymatic steps. First, RNA triphosphatase removes the 5′‐γ‐phosphate group from the first transcribed nucleotide of pre‐mRNA. Next, guanylyltransferase catalyses the transfer of a guanine monophosphate (GMP) nucleotide to the RNA 5′‐diphosphate end, forming a guanosine cap (GpppN) with an inverted 5′–5′ linkage. Finally, RNA (guanine‐N7−) methyltransferase methylates this guanosine cap to produce the 7‐methylguanosine cap (m⁷GpppN).^[7]^ The N7‐methyl guanosine cap (m⁷G) is added co‐transcriptionally to the 5′ end of nascent RNA polymerase II transcripts when they reach a length of 22–25 nucleotides.^[8]^ First discovered five decades ago by Shatkin, Furuichi and Moss, the 5′ m⁷GpppN cap stands as a hallmark feature of eukaryotic mRNA, be it cellular or viral, providing stability to mRNA molecules and fostering their efficient translation.[^9^, ^10^, ^11^, ^12^, ^13^] However, research in the late 1970s revealed that nucleotide metabolites such as nicotinamide adenine dinucleotide (NAD) and flavin adenine dinucleotide (FAD), owing to their adenosine nucleotide components, could serve as initiating nucleotides for RNA synthesis *in vitro* by *Escherichia coli* RNA polymerase.^[14]^ Further investigation along the same lines demonstrated that nucleotide coenzymes, including dephospho‐CoA (dpCoA), NAD and FAD, could initiate transcription by the T7 class II promoters of T7 RNA polymerases *in vitro*.^[15]^ Additionally, bacterial RNA polymerases were observed to initiate transcription using prevalent uridine‐containing cell wall precursors like UDP‐glucose (UDP‐Glc) and UDP−N‐acetylglucosamine (UDP‐GlcNAc),^[16]^ suggesting the potential for nucleotide metabolites to modify the 5′ end of bacterial RNA. These pioneering studies established a new field, non‐canonical 5′ capping and non‐canonical initiating nucleotides (NCINs), which remained largely unexplored until very recently.

Thanks to improved and increasingly sophisticated methodologies, in 2009, liquid chromatography‐mass spectrometry (LC–MS)‐based approaches provided the first evidence that NCINs are indeed integrated into RNA *in vivo*.[^17^, ^18^] The application of these methods has led to the discovery that NAD,^[17]^ dp‐CoA^[18]^ and a series of dp‐CoA derivatives, including succinyl‐, acetyl‐ and methylmalonyl‐dephospho‐CoA, are covalently attached to the 5′ terminus of RNA in *E. coli* and *Streptomyces venezuelae*.[^17^, ^18^] This discovery opened up new directions for advancing our understanding of RNA capping mechanisms.

Later, other metabolite caps, such as FAD, UDP‐Glc and UDP‐GlcNAc, were detected covalently attached to viral and cellular RNA in both prokaryotes and eukaryotes using LC–MS.^[19]^ More recently, FAD was detected at the 5′ end of the hepatitis C virus (HCV) RNA with a frequency of 75 %.^[20]^ A low average frequency of FAD capping has been observed in human mRNA (0.02 %)^[19]^ and in short RNAs.^[21]^

In 2019, an entirely new class of 5′‐RNA caps with the structure of dinucleoside polyphosphates (Np_*n*_Ns) was discovered in *E. coli*.[^22^, ^23^] Recently, the existence of diadenosine tetraphosphate‐ (Ap₄A−) RNA was also reported in human and rat cell lines.^[24]^

Moreover, although proteins have traditionally been considered the primary targets of ADP‐ribosylation, evidence suggests that nucleic acids can also undergo this modification.[^25^, ^26^] Five years ago, it was shown that ADP‐ribosylation can occur on the 5′‐phosphate of RNA *in vitro*, a process catalyzed by various Poly (ADP‐ribose) polymerases (PARPs) and reversible by several hydrolases.^[27]^ Additionally, RNA 2′‐phosphotransferase (Tpt1) from bacteria and fungi, as well as its ortholog in higher organisms, TRPT1, can mediate ADP‐ribosylation of the 5’ phosphorylated end of RNA^[28].^ Further, the occurrence of mono(ADP‐ribosyl)ation (MARylation) of RNA was confirmed *in vivo* using an antibody‐based approach.^[29]^ Additionally, a recent study involving bacteriophage T4 revealed that an ADP‐ribosyltransferase (ART) enzyme, ModB, can use NAD‐RNA as a substrate to attach entire RNA chains to acceptor proteins, forming a unique RNA‐protein conjugate in a process known as “RNAylation”.^[30]^ This finding reveals a direct link between RNA modification and post‐translational protein modification.

The discovery of non‐canonical RNA caps, initially in bacteria^[31]^ and later in other organisms,[^32^, ^33^, ^34^, ^35^, ^36^, ^37^] has sparked a surge in research focusing on the “writers”, “readers”, and “erasers” of these caps. It has been demonstrated that bacterial, yeast, and human mitochondrial RNA polymerases can incorporate nucleotide metabolites during transcription initiation, both *in vitro* and *in vivo*,[^16^, ^38^, ^39^] thus identifying RNA polymerases as the “writers” of these RNA modifications. Recent studies have also revealed that viral RNA‐dependent RNA polymerases can initiate replication with FAD as a NCIN.^[20]^ Post‐transcriptional mechanisms for cap addition have been suggested for NAD as RNA cap of intron‐encoded small nucleolar RNAs (snoRNAs) in humans^[33]^ and plants,[^33^, ^40^] and recently also for yeast cytoplasmic mRNA.^[41]^ Despite these insights, specific enzymes responsible for NAD capping have yet to be identified. Alternative formation of Np_*n*_N‐RNA, involving Lysyl‐tRNA synthetase (LysRS) has been also reported in *E. coli* RNA,^[22]^ although this activity was not confirmed in later studies.^[24]^

The identification of decapping enzymes, or “erasers,” which specifically recognize and remove non‐canonical 5′ caps, is crucial for understanding RNA stability and turnover. Research across various model organisms has identified several NAD decapping (deNADding) enzymes belonging to two distinct enzyme classes. The first class comprises Nudix hydrolases, including NudC in bacteria,[^31^, ^42^, ^43^] and its eukaryotic homologs, Npy1 in yeast^[44]^ and Nudt12[^45^, ^46^] and Nudt16^[46]^ in mammalian cells, which cleave the pyrophosphate bond within the NAD cap to release nicotinamide mononucleotide (NMN) and monophosphorylated RNA (p‐RNA).[^31^, ^45^, ^47^] The second class includes enzymes from the DXO/Rai1 family, which, unlike Nudix proteins, target the phosphodiester bond to produce p‐RNAs while releasing an intact NAD molecule.[^33^, ^48^, ^49^, ^50^] Notably, homologs of DXO/Rai1 have not been identified in prokaryotes. Both enzyme classes have also been shown to act on other non‐canonical capped RNAs, removing FAD (deFADding) and dpCoA (deCoAping) from RNA.^[21]^ The activity of mammalian Nudix enzymes and those from *Arabidopsis thaliana* toward non‐canonical caps has been thoroughly characterized through biochemical analyses.[^46^, ^51^] Another bacterial Nudix hydrolase, RppH, removes Np_4_N^[22]^ and Np_*n*_N caps, with its activity increasing with the number of bridging phosphates.^[52]^ The ApaH enzyme, a member of another family ‐ ApaH‐like phosphatase, also cleave Np_*n*_N caps.[^22^, ^52^]

Recently, new types of potential non‐canonical cap “erasers” were reported. The human glycohydrolase CD38 was shown to convert NAD‐RNA into 5’‐ADP‐ribose‐RNA by releasing nicotinamide (NAM) *in vitro*.^[53]^ This mechanism indicates that the ADP‐ribose RNA produced by CD38 arises from the modification of an existing RNA molecule, in contrast to TRPT1, which catalyzes the transfer of ADP‐ribose from NAD to the RNA substrate.^[28]^ Although its *in vivo* decapping activity remains unverified, CD38 introduces a potential new deNADding pathway for NAD‐RNA. It is important to mention that the structure of ADP‐ribosylated RNA can differ depending on the mechanism of its formation. While Tpt1 was proposed to attach the 5’ phosphate of RNA (DNA) to the 1’ carbon of ribose, the ADP‐ribose‐RNA formed by CD38 has a free 1’ carbon, and the RNA is connected to ADP‐ribose via a classical 3’‐5’ phosphodiester bond. Additionally, Toll/interleukin‐1 receptor (TIR) domain‐containing proteins from various bacterial species and one archaeal species have been identified as NAD‐RNA deNAMing enzymes, capable of removing the NAM moiety from NAD‐RNAs.^[54]^

Characterization of NAD and FAD cap‐binding proteins in budding yeast has led to the identification of the highly conserved Xrn1 and Rat1 5′‐3′ exoribonucleases as novel deNADding^[55]^ and deFADding^[56]^ enzymes, respectively. Human Xrn1 has also been shown to hydrolyze NAD‐ and FAD‐capped RNAs. The recently discovered bacterial 5′‐3′ exoribonuclease RNase AM (yciV) in *E. coli*[^57^, ^58^] exhibits deFADding activity in *E. coli* cells.^[56]^ These studies, which focused on identifying cellular proteins interacting with non‐canonical caps using cap RNA affinity purification and mass spectrometry, primarily revealed RNA nucleases.[^55^, ^56^] Therefore, further research is needed to identify *bona fide* non‐canonical cap‐binding proteins – i. e. readers of these modifications.

Although significant progress has been made in the research and identification of non‐canonical caps and their associated “writers”, “readers”, and “erasers”, there is an urgent need to develop new sequencing techniques to characterize the RNA types bearing these caps and to assess their functional roles in living systems. While the biosynthesis, biodegradation, and potential roles of non‐canonical RNA caps have been reviewed elsewhere,^[59]^ this review will focus on the most emblematic and decisive identification, capturing, and profiling techniques developed to date. It will assess the advantages and limitations of these methods and emphasize the need for sophisticated and reliable profiling techniques to enhance the functional analysis of non‐canonical capped RNAs.

## 2 Detection Techniques

### 2.1 Mass Spectrometry‐Based Methods for the Detection of Non‐Canonical Caps

**Figure 1:**
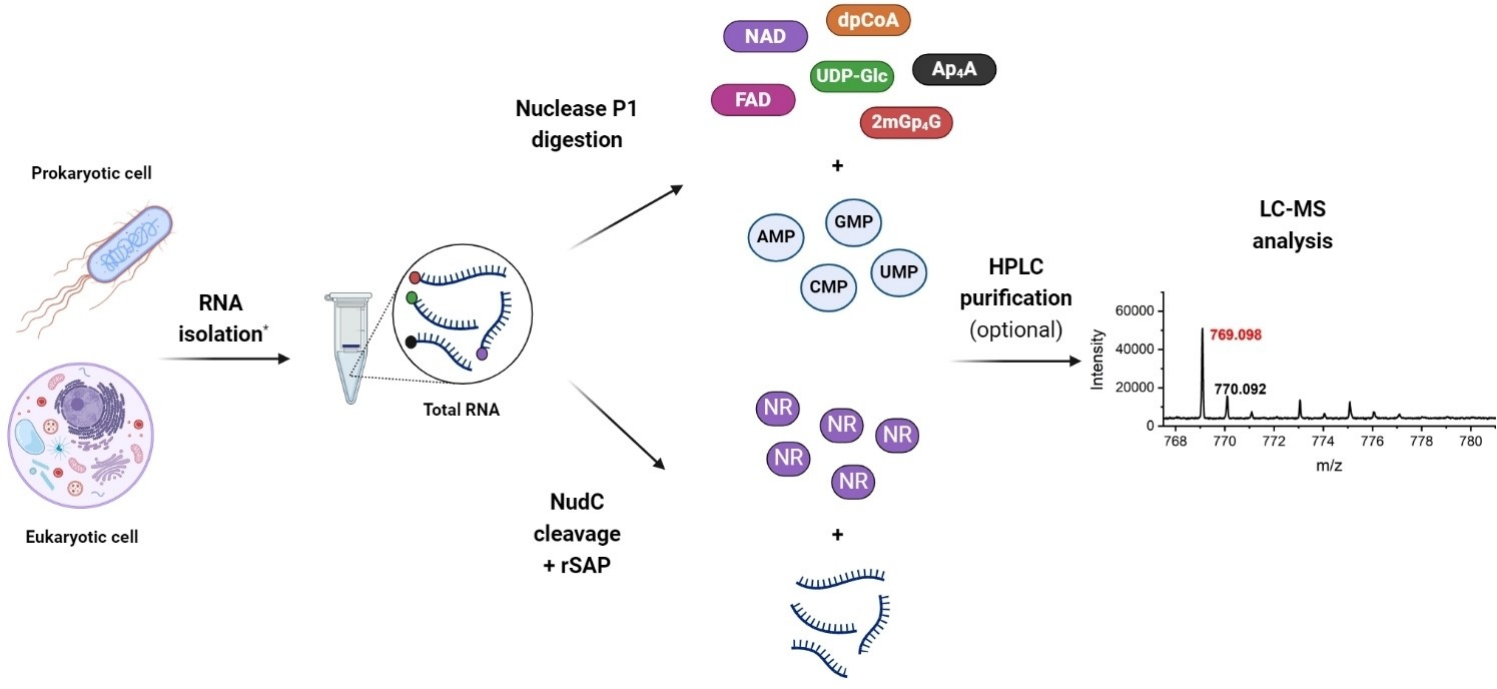
Detection of non‐canonical capped RNAs using LC–MS techniques. RNA is isolated from either prokaryotic or eukaryotic cells using extraction kits (*e. g., TRIzol, RNAzol, QIAzol). The purified total RNA (top) is digested by Nuclease P1 into nucleotides. Alternatively, sRNA (bottom) is treated with the NudC enzyme to cleave nicotinamide mononucleotide (NMN) from NAD‐capped RNA. The NMN is then converted to nicotinamide riboside (NR) using shrimp alkaline phosphatase (rSAP). The fraction of small molecules is finally analyzed by LC–MS. An HPLC purification step can be added prior to LC–MS. The figure was created using BioRender.com.

The first method for detecting NAD, CoA and other CoA thioesters as covalent conjugates to cellular RNA in bacterial cells was reported in 2009.[^17^, ^18^] Generally, the LC–MS method for identifying non‐canonical 5′ RNA caps involves several key steps: first, enriching the small RNA fraction through size‐exclusion chromatography; second, enzymatic hydrolysis of the isolated small RNA fraction; and finally, detecting the NAD or small molecule‐nucleotide conjugates cleaved from the RNA using high‐resolution LC–MS analysis. This approach entails meticulously isolating highly purified total RNA, poly(A)+ RNA, or size‐fractionated RNA. An important step is separating them from non‐covalently bound molecules and digesting the RNA into nucleotides using nuclease P1.[^17^, ^18^] Notably, the chemical bonds associated with 5′ capping (pyrophosphate or phosphate anhydride) are resilient to the enzyme's phosphodiesterase activity, preserving the 5′ non‐canonical moieties. These intact moieties are then subjected to qualitative and quantitative analysis using LC–MS/MS (Figure 1). Overall, MS‐based methods offer a powerful and versatile approach for detecting various types of RNA modifications and non‐canonical caps. They provide also high sensitivity and specificity, enabling the accurate identification and characterization of low‐abundant non‐canonical caps even in complex RNA mixtures. However, these methods have also limitations. Despite their high sensitivity, MS may not detect all non‐canonical caps, especially those present at very low levels, sensitive to used technique or those not easily ionized. Additionally, fragmentation of non‐canonical caps during MS analysis can complicate data interpretation, particularly when structurally complex non‐canonical caps are concerned or when distinguishing between different isomeric forms. The delicate nature and high cost of mass spectrometry equipment necessitate careful sample preparation. Preparing samples for MS analysis of RNA can be laborious and may require specialized techniques to isolate and purify RNA samples. It is important to mention that the preparation of RNA samples for the detection of NAD‐capped RNA requires specific treatment. To accurately determine the amount of NAD covalently linked to RNA, a urea wash must be included to remove non‐covalently bound NAD.[^44^, ^60^, ^61^] Omitting this step leads to overestimated values that do not accurately reflect the actual amounts. This issue should be addressed when the RNA cap is present in its free form within the cell, such as in 5′‐CoA or FAD. However, the positive charge of NAD may result in a stronger affinity for the negatively charged RNA backbone compared to other molecules.

The evidence for the existence of FAD, UDP‐Glc and UDP‐GlcNAc as 5′ RNA caps *in vivo* emerged ten years after the discovery of NAD^[17]^ and CoA caps,^[18]^ thanks to the development of a systems‐level MS‐based technique called CapQuant.^[19]^ This method combines offline HPLC enrichment of cap nucleotides with isotope‐dilution LC–MS/MS analysis, enabling the absolute quantification of RNA caps (Figure 1).^[19]^ The additional HPLC step, following Nuclease P1 digestion, makes CapQuant a targeted MS approach with predefined mass transitions corresponding to known non‐canonical caps. This step enhances detection specificity and sensitivity towards the RNA cap landscape, reaching attomole levels per microgram of RNA analysed.^[19]^ However, the method's target specificity can be a double‐edged sword, as it may not be as effective in detecting new or unknown types of RNA caps that have not been specifically targeted. The CapQuant was applied to RNA from purified dengue virus, *E. coli*, *Saccharomyces cerevisiae*, mouse tissues and human cells.^[19]^

The structural similarity between Np_*n*_Ns and the m⁷G RNA cap, along with the demonstration that Np_*n*_Ns can serve as NCINs for RNA polymerase during *in vitro* transcription^[52]^ similarly to NAD and CoA,^[38]^ has led to the hypothesis that Np_*n*_Ns might exist as 5′‐RNA caps *in vivo*. Two independent studies concurrently reported the presence of these RNA caps in bacteria. One study employed LC–MS techniques^[23]^ whereas the other purely relied on electrophoretic analysis.^[22]^ The Cahova laboratory provided evidence of their existence as RNA caps in *E. coli* using an LC–MS technique.[^23^, ^52^] The focus was on short RNA (sRNA), where the NAD RNA cap^[17]^ and the CoA RNA cap^[18]^ had also been detected. To ensure the accuracy of the results, the purified sRNA was washed to remove all non‐covalently interacting molecules and then digested with Nuclease P1 into nucleotides.^[52]^ Negative control samples that omitted Nuclease P1 showed no signals of nucleotides or Np_*n*_Ns, ruling out non‐covalent contamination.^[52]^ The researchers identified nine new RNA caps ‐ Ap_3_A, Ap_3_G, Ap_5_A, m^7^Gp_4_Gm, mAp_5_G, m^6^Ap_3_A, 2mAp_5_G, mAp_4_G, and mAp_5_A ‐, six of which were mono‐ or dimethylated.^[52]^ Given that intracellular concentrations of Np_*n*_Ns are known to increase under stress conditions,[^62^, ^63^] cells were collected from both exponential and late stationary phases of growth. A significant increase in Np_*n*_Ns‐capped RNA was observed in the late stationary phase.^[52]^ This discovery, combined with the finding that the highest amount of methylated Np_*n*_Ns caps occurs in the stationary phase,^[52]^ suggests that bacteria use methylated caps to stabilize some RNA under starvation conditions, likely to prioritize the expression of genes needed to cope with cellular stress.

The existence of these Np_*n*_Ns‐RNA caps has also been proven in eukaryotes, both in human and rat cell lines.^[24]^ Confirmation of the presence of Ap₄A as a NCIN was achieved through the development of a highly sensitive LC–MS method, specifically using a triple quadrupole (QQQ LC–MS)^[24]^. Further evaluation revealed that Ap₄A‐RNA is not recognized by eIF4E and it is not translated *in vitro* using rabbit reticulocyte nor in cells after transfection. Nevertheless, it is recognized as self by the cell, and it does not trigger an immune response,^[24]^ which indicates it is a natural component of the transcriptome. Although there are several clues suggesting a potential role for Ap₄A‐RNA in the cell, current evidence is insufficient to state its function with certainty.

### 2.2 Spectroscopy‐Based Assays

**Figure 2:**
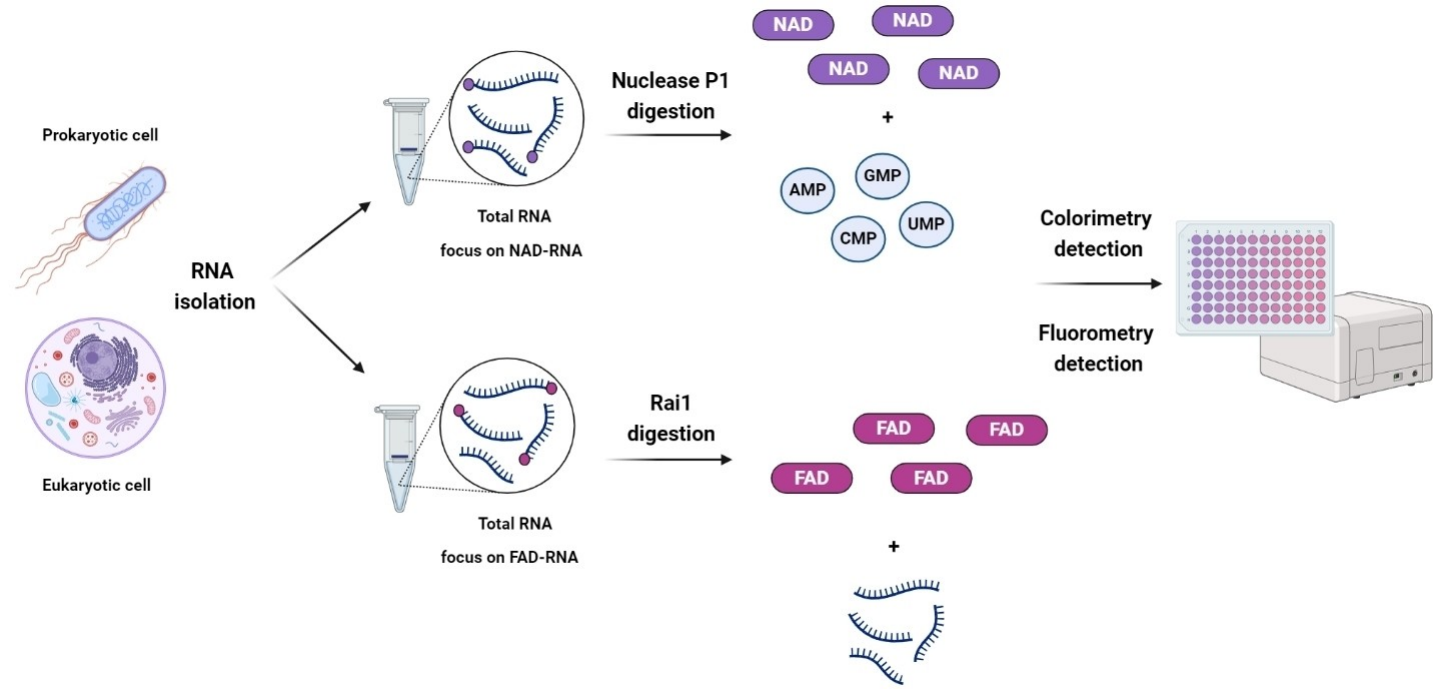
Detection of NAD/FAD‐RNA by spectroscopy‐based assays. After RNA isolation, the RNA is treated with Nuclease P1 (top) or Rai1 (bottom) to release NAD and FAD, respectively. The released 5′ NAD/FAD is then quantified using a commercially available colorimetric/fluorometric assay. The figure was created using BioRender.com.

An alternative method to LC–MS detection is based on colorimetry. NAD cap detection and quantitation (NAD‐capQ)^[64]^ is a technique for measuring and quantifying NAD caps in cells. The NAD‐capQ method is built upon the method pioneered by Chen and colleagues,^[17]^ substituting MS analysis with a flexible colorimetric assay. This approach involves a two‐step process: first, RNA is enzymatically treated with nuclease P1 to break phosphodiester bonds and release 5′ NAD. The released 5′ NAD is then quantified using a commercially available colorimetric assay (Figure 2, top). This assay relies on an enzymatic cycling reaction where NAD is converted to NADH, which subsequently reacts with a probe to generate a product that absorbs light at a wavelength of 450 nm, measurable by spectrophotometry.^[64]^

An analogous method, known as FAD‐capQ, was subsequently developed to detect and quantify FAD‐capped RNAs in human cells.^[21]^ Similar to NAD‐capQ,^[64]^ this approach follows a two‐step procedure: FAD‐capped RNA is initially treated with *Schizosaccharomyces pombe* Rai1 (SpRai1), which is capable of removing dpCoA and FAD caps *in vitro*.^[65]^ SpRai1 cleaves the phosphodiester bond between the first and second encoded nucleotides in the RNA, releasing intact 5′ FAD. Subsequently, the released FAD is quantified using a commercially available fluorometric assay specific for FAD (Figure 2, bottom). This assay relies on FAD acting as a crucial cofactor in an oxidase reaction, which produces a corresponding fluorescent emission detected by the OxiRed probe (Ex/Em=535/587). The parameters of the fluorometric assay allow for the detection of FAD in the femtomole (fmol) range.^[21]^ FAD‐capQ differs from the NAD‐capQ assay^[64]^ in two main aspects: firstly, SpRai1 is employed in the FAD‐capQ assay instead of Nuclease P1, which was found to affect the fluorescence intensity of the OxiRed probe over the course of the reaction. Secondly, a lower amount (3 μg) of RNA is used in the FAD‐capQ assay due to the observation that higher RNA quantities (50 μg) tend to quench the probe signal. This study identified FAD‐capped RNAs in both poly(A)+ and short RNAs (less than ∼200 nucleotides) in mammalian cells.

Typically, the sensitivity of LC–MS/MS, which achieves detection limits in the femtomolar (fM) to low picomolar (pM) range is higher compared to spectroscopy‐based assays (femtomolar to micromolar (μM) concentrations). This difference in sensitivity may restrict the applicability of spectroscopy‐based methods for detecting non‐canonical caps, particularly when these modifications are present in low concentration. However, spectroscopy‐based techniques can be advantageous in scenarios where a simpler and faster analysis is required. Moreover, LC–MS/MS not only detects these caps with higher sensitivity but also provides detailed structural characterization, thereby establishing itself as the gold standard for the detection and quantification of non‐canonical RNA caps.

### 2.3 Bioenzymatic Assay

**Figure 3:**
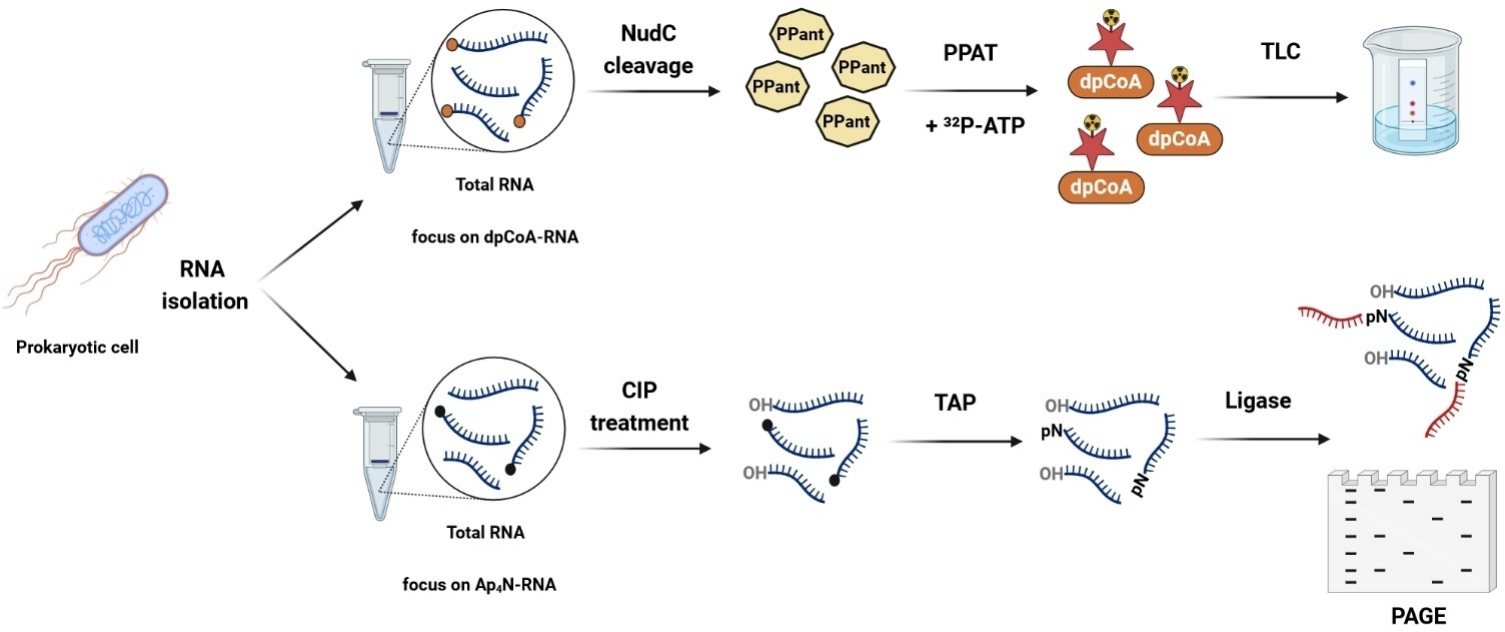
Detection of CoA/dpCoA‐RNA and Ap_4_N‐RNA by bioenzymatic assay. (Top) Isolated RNA is cleaved by NudC to convert CoA or dpCoA‐RNA into phosphopantetheine (PPant). Phosphopantetheine adenylyltransferase (PPAT) then converts PPant to dpCoA using [α‐^32^P]‐ATP. The conversion to ^32^P‐labeled dpCoA is visualized through thin‐layer chromatography (TLC). (Bottom) Isolated RNA is treated with Calf Intestinal Alkaline Phosphatase (CIP), converting 5′ p‐RNA into OH‐RNA while leaving Np_4_A intact. Tobacco Acid Pyrophosphatase (TAP) cleaves Ap_4_N‐RNA, leaving pN‐RNA for ligation. Ligated forms are visualized by Polyacrylamide Gel Electrophoresis (PAGE). The figure was created using BioRender.com.

Although evidence for CoA and its derivative forms as non‐canonical RNA cap^[18]^ emerged around the same time as for NAD,^[17]^ their low abundance makes the identification of particular CoA‐RNA transcripts challenging. Evidence for dpCoA‐RNAs was initially limited to the two bacterial species in which they were discovered.^[18]^ The Jäschke group developed a bi‐enzymatic assay to detect and quantify CoA and dpCoA‐RNA in the small RNA fraction of the firmicute *Staphylococcus aureus*,^[66]^ an organism with high concentration of CoA (658.2 nmol per gram of cell dry weight) and dpCoA (155.6 nmol per gram of cell dry weight).^[67]^ This assay utilizes *E. coli* NudiX hydrolase NudC to convert CoA or dpCoA‐RNA into phosphopantetheine (PPant). *E. coli* phosphopantetheine adenylyltransferase (PPAT) then converts PPant to dpCoA using ATP. By incorporating [α‐^32^P]‐ATP, they generated ^32^P‐labelled dpCoA and visualized its conversion through thin‐layer chromatography (TLC) (Figure 3, top). The assay accurately quantified CoA and dpCoA‐RNA in the range of 50–1000 fmol and did not detect acetyl‐CoA, indicating specificity for CoA, dpCoA‐RNA and not their ester forms. This enzymatic assay allows for the simultaneous analysis of multiple samples with sensitivity comparable to LC–MS,[^19^, ^68^, ^69^] but it does not provide information about the sequence of dpCoA‐RNA.

Simultaneously to LC–MS detection of Np_*n*_Ns RNA caps in bacteria,^[23]^ the Belasco laboratory demonstrated that under disulfide stress conditions —where dinucleoside tetraphosphate (Np₄Ns) concentrations rise significantly (220–950 μM)[^70^, ^71^] compared to their levels in unstressed cells (0.6–2.4 μM)^[72]^ — certain *E. coli* mRNAs and sRNAs acquire a cognate Ap₄N caps.^[22]^ The presence of this cap‐like structure at the 5′ end was investigated through *in vitro* testing. First, the resistance of 5′‐terminal phosphates of mRNA from cadmium‐stressed cells to removal by alkaline phosphatase was assessed, followed by their ability to be converted into a ligatable form after pyrophosphatase treatment (Figure 3, bottom).[^22^, ^73^, ^74^] Secondly, the reduced electrophoretic mobility of cellular transcripts was examined using a matrix that selectively retards capped RNAs.^[22]^ Specifically, a polyacrylamide gel containing boronate side chains was used to reduce the electrophoretic mobility of transcripts bearing a vicinal diol, a characteristic feature of most 5′ caps,^[75]^ enabling the separation of capped from uncapped RNAs. The RNA transcripts were subsequently detected by Northern blot analysis.^[22]^

### 2.4 Antibody‐Based Approach

**Figure 4:**
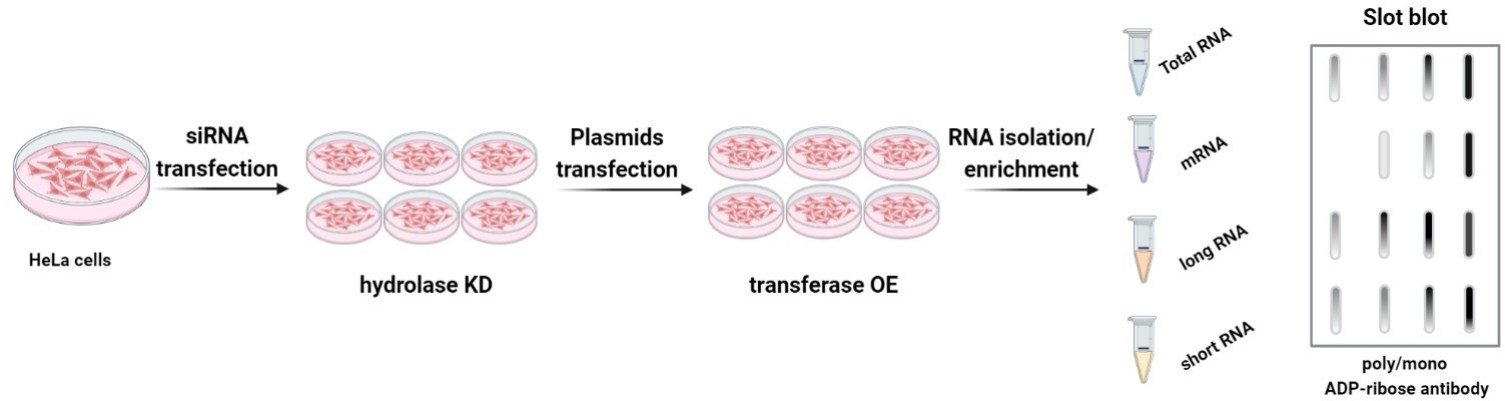
Detection of ADPr‐RNA by immunoassay. HeLa cells undergo siRNA transfection to knock down hydrolases (KD), followed by transfection with plasmids overexpressing (OE) the transferase. Total, long, and small RNA pools are extracted from cell lysates, and mRNA is enriched from the total RNA fraction using oligo(dT) beads. RNA samples are subsequently blotted, and ADP‐ribosylation of the blotted RNAs is detected using a poly/mono ADP‐ribose antibody. The figure was created using BioRender.com.

Following the *in vitro* discovery of ADP‐ribosylated RNA (ADPr‐RNA),^[27]^ subsequent evidence demonstrated that various RNA species undergo also ADP‐ribosylation *in vivo* within human cells.^[29]^ To detect endogenous ADPr‐RNA, they used a commercially available poly/mono‐ADP‐ribose antibody.^[29]^ Given the challenges in detection, they hypothesized that RNA ADP‐ribosylation is highly dynamic and efficiently reversed by cellular hydrolases. Therefore, to identify cellular ADPr‐RNA, they overexpressed TRPT1, a human transferase known to ADP‐ribosylate RNA *in vitro*.[^27^, ^28^] Additionally, they silenced the hydrolases PARG, TARG1, and ARH3 individually or in combination to reduce the turnover of ADPr‐RNA. From the transfected cells, they isolated total RNA and used slot blotting to detect ADPr‐RNA (Figure 4). The combination of TRPT1 overexpression and triple knockdown of hydrolases resulted in the most substantial increase of ADPr‐RNA in cells.^[29]^ Treatment of cellular ADPr‐RNA with DNase I or proteinase K did not eliminate the signal, ruling out contamination from ADP‐ribosylated DNA or protein.^[29]^ By employing this combinatorial approach across various cellular RNA pools (including total RNA, long RNA, short RNA, and mRNA), they demonstrated significant variability in ADP‐ribosylation patterns, indicating that different enzymes exhibit preferences for modifying distinct RNA species.^[29]^ Generally, the use of antibodies to detect small molecules is limited by off‐target effects. To overcome this problem, they generated ADPr‐capped and structurally similar NAD‐capped RNA for comparison with non‐capped RNA and the auto‐modified catalytic domain of PARP10. The antibody successfully detected auto‐modified and purified ADPr‐RNA, while showing no reactivity towards non‐capped RNA or NAD‐RNA.^[29]^ Moreover, the antibody‐based approach provides only semi‐quantitative results. Future analyses will require more quantitative methods to assess the specific contributions of individual enzymes to ADPr‐capping of RNA, along with sequencing techniques to identify ADPr‐RNA.

## 3 Sequencing Methods for the Identification of Capped RNA

**Figure 5:**
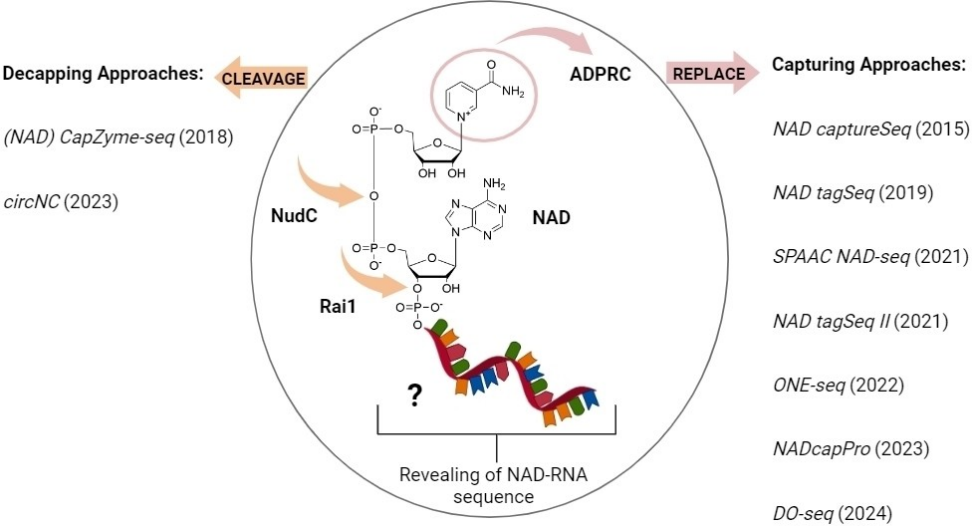
All known sequencing methods to date (2015–2024) for the identification of NAD‐capped RNAs. These methods include two main approaches: decapping approaches (on the left), which use decapping enzymes (e. g., NudC and Rai1) to selectively remove and target the cap of interest; and capturing approaches (on the right), which involve the enzymatic modification of the NAD cap using the ADPRC enzyme with primary alcohol substrates. The figure was created using BioRender.com.

The biggest limitation of the aforementioned methods[^17^, ^18^, ^21^, ^22^, ^29^, ^52^, ^64^, ^66^] is that they do not provide information about the identity of the RNA bearing the specific cap. NAD, as a non‐canonical cap, is the most studied and characterized. The development of numerous NAD detection and profiling techniques over the years has laid the groundwork for investigating the regulatory roles of the NAD cap in RNA synthesis, stability, and degradation (Figure 5). Recently, a thorough review on all NAD‐RNA identification and sequencing techniques was published, giving detailed insights and comprehensive information on this topic.^[76]^ For the sake of completeness and to inspire the development of sequencing techniques for other non‐canonical RNA caps, we consider it important to discuss these methods here as well.

### 3.1 NAD CaptureSeq

A milestone in the study and characterization of NAD‐RNAs, which laid the foundation for the development of numerous and increasingly sophisticated NAD techniques, was achieved in 2015 by Jäschke laboratory.^[31]^ This method, named NAD captureSeq (NAD capSeq), combines enzymatic reaction with click‐chemistry and with high‐throughput sequencing platforms, enabling comprehensive analysis of NAD‐capped RNAs *in vivo*.^[31]^ The peculiarity of NAD capSeq lies in its exploitation of a special characteristic of the *Aplysia californica* ADP‐ribosyl cyclase (ADPRC).^[77]^ This enzyme was reported to catalyse transglycosylation reactions, where nicotinamide in NAD is replaced with N‐heterocyclic nucleophiles.^[78]^ In the NAD capSeq method, ADPRC is used to perform transglycosylation reactions on RNA molecules with NAD caps. During this process, nicotinamide is substituted with alkynyl alcohols, which serve as ”clickable” moieties for further chemical modifications. Following the transglycosylation reaction, the alkynyl group introduced to the RNA molecule can be conjugated to a biotin‐azide using a copper‐catalyzed cycloaddition reaction (CuAAC).[^79^, ^80^] This step labels the RNA molecules with biotin, facilitating their enrichment and subsequent analysis. The 5′‐biotinylated RNA transcripts are then isolated and enriched using streptavidin beads or other biotin‐binding reagents, aiding in the purification and concentration of the RNA molecules of interest. Finally, the enriched RNAs are used to produce a cDNA library for the identification and quantification of NAD‐RNAs using high‐throughput sequencing.^[31]^ This method provides comprehensive information about the RNA population and the distribution of NAD caps across different transcripts.^[31]^ The method mainly compensates for the lack of sequence information associated with the LC–MS method and enables the identification of specific RNA types capped with NAD, opening new opportunities to study NAD‐RNA. Initially applied to *E. coli*, this method revealed that primarily certain small regulatory RNAs and 5′‐fragments of different mRNAs are capped with NAD,^[31]^ playing roles in cellular metabolism[^81^, ^82^] and stress responses.[^83^, ^84^, ^85^, ^86^, ^87^] Subsequent studies have also revealed NAD capping in mRNA from the budding yeast *S. cerevisiae*,^[32]^ as well as in human cells,^[33]^ plants[^34^, ^35^] and archaea.[^36^, ^37^] These findings indicate a wide range of NAD‐capped RNAs not only in prokaryotes, but also across the eukaryotic transcriptome. However, NAD captureSeq, despite being a robust and innovative method for the identification of NAD‐RNAs that has greatly expanded our knowledge of them, presents limitations. Firstly, the method relies on copper ions as a catalyst for the cycloaddition reaction, which is known to potentially induce RNA fragmentation. However, if used correctly in combination with copper‐stabilizing agents, this issue can be mitigated. This, combined with sequencing library construction, can lead to the loss of information regarding the overall sequence structures of NAD‐RNAs and could impede the detection of less abundant RNAs. Secondly, the promiscuity of the ADPRC enzyme poses a challenge. Particularly in eukaryotes, the residual activity of ADPRC on m⁷G‐capped RNAs can limit the specificity of NAD captureSeq in profiling NAD‐capped RNAs.

### 3.2 CapZyme‐Seq

Few years later, an alternative method, known as CapZyme‐seq,^[65]^ was proposed for the analysis of NAD‐RNAs and other non‐canonically capped RNAs. This enzymatic approach relies on the selectivity of decapping enzymes. The RNA extracts are selectively targeted by an NAD decapping enzyme, like NudC[^31^, ^42^] and Rai1,^[33]^ resulting in the production of 5′ p‐RNA. p‐RNA serves as a suitable substrate for the ligation of single‐stranded oligonucleotide adaptors required for subsequent library construction and high‐throughput sequencing. CapZyme‐seq integrates selective enzymatic treatment of both non‐canonical capped and uncapped 5′ ends with high‐throughput sequencing, enabling precise identification of RNA 5′ ends at single‐nucleotide resolution.^[65]^ Additionally, it facilitates the quantification of the relative abundance of NAD‐capped and uncapped RNA molecules.^[65]^ However, a significant shortcoming of this method, which considerably restricts its versatility, lies in the overlapping specificity of most decapping enzymes. This results in the identification of non‐canonically capped RNA in general, without distinguishing between NAD caps and others. In their work aimed at selectively processing NAD‐capped RNA to 5′‐monophosphate RNA,^[65]^ researchers employed either the bacterial NudiX pyrophosphatase NudC[^31^, ^42^] or the fungal RNA‐decapping enzyme DXO/Rai1.^[33]^ Rai1 demonstrates robust deNADding activity and lacks decapping activity on m⁷G‐capped RNA.[^33^, ^88^, ^89^] However, it is worth noting Rai1′s capability to hydrolyse a range of non‐canonical caps (NAD, FAD, dpCoA, and GlcNAc).[^21^, ^33^, ^65^] Additionally, subsequent studies on NudC revealed its activity on canonical m⁷G‐capped RNA[^45^, ^90^] and dpCoA‐capped RNA,[^38^, ^91^] rendering it unsuitable for NAD caps.

### 3.3 NAD TagSeq and NAD TagSeq II

In 2019, Zhang et al. developed a method similar to NAD captureSeq,^[31]^ called NAD tagSeq.^[35]^ The initial step closely mirrors that of NAD captureSeq.^[31]^ However, instead of employing biotinazide for cycloaddition after the ADPRC reaction, it involves the cycloaddition of alkyne‐functionalized NAD‐RNA to a synthetic RNA (tagRNA) via an azide group at its 3′ end. These tagged RNAs are then enriched with biotinylated DNA complementary to the tagRNA and subsequently sequenced directly using single‐molecule RNA sequencing by the Oxford Nanopore Technology (ONT). Therefore, this approach offers the potential to overcome nonspecific binding of RNAs to the streptavidin resin, which can lead to false positives and reduced sensitivity.^[31]^ Moreover, single‐molecule RNA sequencing enables the revelation of the sequences of entire NAD‐RNA transcripts. NAD tagSeq findings in *Arabidopsis* revealed that NAD‐capped RNAs primarily originate from protein‐coding genes and exhibit fundamentally similar sequence structures to those of conventional m⁷G‐capped RNAs.^[35]^

However, the NAD tagSeq^[35]^ as well as the NAD captureSeq,^[31]^ rely on CuAAC click chemistry to label NAD‐RNAs. The risk of RNA fragmentation and/or degradation therefore persists, potentially leading to reduced sensitivity, loss of complete sequence information, and underestimation of NAD‐capped transcripts. To address these limitations, the same group developed a second version, termed NAD tagSeq II.^[92]^ This modified method employs copper‐free strain‐promoted azide‐alkyne cycloaddition (SPAAC) for tagging NAD‐RNAs. SPAAC tagging results in longer reads and significantly higher efficiency in identifying NAD‐RNAs compared to CuAAC tagging.^[92]^ NAD tagSeq II was used to compare NAD‐RNA and total transcriptome profiles in *E. coli* cells during exponential and stationary growth phases. The study revealed genome‐wide alterations in NAD‐RNA profiles across different growth stages.^[92]^ However, in both NAD tagSeq versions,[^35^, ^92^] the CuAAC and SPAAC reactions create a junction between the NAD cap and the RNA tag that is not typical of a nucleotide. This structural anomaly causes inaccurate base calling of the surrounding nucleotides by the standard ONT algorithm, complicating the determination of precise 5′ ends of NAD‐RNAs. Developing machine learning algorithms will be necessary for accurate base calling around these junctions and, consequently, the cap structure.

### 3.4 SPAAC‐NAD‐Seq

Whereas NAD captureSeq employs 4‐pentyn‐1‐ol,^[31]^ SPAAC‐NAD‐seq^[93]^ uses 3‐azido‐1‐propanol for the ADPRC‐catalysed transglycosylation reaction, resulting in the generation of an azide group to replace the nicotinamide moiety. The introduced azide moiety undergoes a SPAAC reaction and can subsequently react with the autocatalytic alkyne moiety of biotin‐PEG4‐dibenzylcyclooctyne, thereby eliminating the need for copper ions.^[93]^ This metal ion‐free SPAAC reaction theoretically enriches NAD‐RNAs while preventing RNA fragmentation, thereby preserving the sequence and structural integrity of NAD‐RNAs. To address the issue of ADPRC reactivity towards canonically capped RNA by both CuAAC (NAD captureSeq^[31]^) and SPAAC‐NAD (SPAAC‐NAD‐seq),^[93]^ an m⁷G‐capped RNA depletion step using an m⁷G antibody was introduced.^[93]^ Immunoprecipitation depletion of m⁷G‐capped RNAs is crucial, especially in eukaryotes, where the RNA pool contains a substantial proportion of m⁷G‐capped RNAs. This step ensures the accurate profiling of NAD‐RNA, preventing false positive hits. Thus, SPAAC‐NAD‐seq can be considered an optimized version of NAD captureSeq.^[31]^ By reducing competition from m⁷G‐capped RNAs, a significant number of *bona fide* NAD‐RNA‐producing genes from *Arabidopsis* have been identified.^[93]^

### 3.5 NADcapPro and CircNC

One of the most recent advancements in NAD‐capturing techniques comes from the Kiledjian laboratory.^[41]^ This approach is designed to circumvent the limitations of previously mentioned methods by combining two orthogonal approaches to more accurately identify eukaryotic NAD‐RNAs. The first method, NAD cap profiling (NADcapPro) method,^[41]^ builds upon previous work using copper‐free SPAAC to minimize RNA fragmentation and to precisely identify eukaryotic NAD‐capped transcripts.^[93]^ Nevertheless, NADcapPro method avoids the need for antibody depletion to reduce m⁷G‐RNA contamination^[93]^ by employing the robust m⁷G decapping activity of yDcps^[94]^ before the SPAAC reaction. yDcps, the yeast scavenger decapping enzyme, effectively eliminates unwanted residual ADPRC activity towards canonical m⁷G caps with its strong pyrophosphatase activity on m⁷G‐capped RNA.^[41]^ Another important modification involves the direct visualization of NAD‐RNAs using streptavidin‐conjugated near‐infrared (IR) IRDye, with sensitivity in the fmol range.^[41]^ NADcapPro provides a robust method to capture high‐ and low‐abundance NAD transcripts in eukaryotes, which are otherwise not detected with standard NAD‐RNA isolation approaches. The second independent orthogonal approach, termed intramolecular circularization of non‐canonical capped RNA (circNC),^[41]^ was developed to map the precise NAD metabolite nucleotide addition site within transcripts validated by NADcapPro to possess NAD caps. The circNC method expands on two previously established techniques, CapZymeSeq^[65]^ and RNA end circularization.^[95]^ This approach relies on the robust *in vitro* deNADding activity of Rai1, which selectively removes the intact NAD moiety from the 5′ end of an RNA molecule, resulting in a 5′ monophosphate RNA structure.^[33]^ Importantly, Rai1 does not cleave m⁷G‐capped transcripts. The monophosphate RNA is circularized intramolecularly using T4 RNA ligase, producing a circular RNA molecule with the 5′ untranslated regions (UTR) fused to the 3′ end poly‐A tail, followed by the 3′ UTR region. Using transcript‐specific primers, cDNA synthesis can be initiated, followed by targeted PCR amplification across the junction.^[41]^ This method provides a mechanism for detecting non‐canonical capped RNAs with nucleotide‐level precision, enabling the precise mapping of cap addition sites as well as the exact 3′ termini of the RNA. Therefore, this method can also serve as an additional control to verify the results of NAD‐sequencing technologies. Additionally, all examined transcripts were polyadenylated, indicating that NAD transcripts are complete, full‐length mRNAs.^[41]^ It is worth noting that Rai1 can also decap FAD and dephospho CoA caps.^[21]^ Therefore, although the RNAs used in the circNC analysis were confirmed by NADcapPro to have an NAD cap, there remains the possibility of detecting a mixture of non‐canonical caps.

### 3.6 ONE‐Seq and DO‐Seq

Another recently published approach proposes a less laborious and faster protocol for identifying NAD‐RNAs directly in total RNA. Unlike previous methods that require multiple reactions, ONE‐seq involves a one‐step chemo‐enzymatic reaction that directly conjugates NAD‐RNA with a biotin affinity tag.^[96]^ They used N‐[2‐(2‐hydroxyethoxy) ethyl]biotinamide (HEEB) as the reactant. HEEB has both a terminal hydroxyl group as the nucleophile and a biotin group as the affinity tag, allowing for the biotinylation of NAD‐capped RNAs in a single step. In the presence of ADPRC, the nicotinamide moiety of NAD can be replaced by HEEB through a nucleophilic reaction, resulting in simultaneous biotinylation. The biotinylated NAD‐RNAs can then be enriched using streptavidin beads. A subsequent NudC‐catalysed cleavage step elutes NAD‐capped RNA from the streptavidin beads, while contaminating m⁷G‐capped RNAs that also react with HEEB remain bound to the beads. The eluted RNAs can then be used for high‐throughput sequencing (epitranscriptomic‐wide profiling) and gene‐specific qRT‐PCR analysis.^[96]^ False positive hits coming from m^7^G‐capped RNA in NAD‐RNA sequencing analysis are a common issue with most current techniques. Remedies such as introducing an antibody‐based pre‐treatment to deplete m⁷G‐capped RNA from purified mRNA are labour‐intensive and require high RNA input,^[93]^ making them unsuitable for gene‐specific analysis. Using NudC‐based post‐treatment to recover NAD‐capped RNAs allows the assay to be performed directly from total RNA with significantly less input compared to other methods.^[96]^ However, studies have reported non‐specific activity of NudC on m^7^G‐capped RNA as well.[^45^, ^90^] Therefore, a new method called DO‐seq has been introduced recently.[^96^, ^97^] This method is a variation of ONE‐seq with one more extra step, the use of yDcps for the cleavage of m^7^G‐capped RNA. DO‐seq was used for mapping NAD‐RNA in four different developmental stages of *Drosophila melanogaster*.

Despite the study of NAD‐RNAs in eukaryotes being in a premature state, a round‐up of these techniques and the information drawn from them have provided a huge push forward. These latest techniques are a testament to how the combination of various ideas can lead to the development of powerful and precise methods, even in more complex organisms. The importance of accurately identifying NAD‐capped RNAs and understanding their metabolism is directly proportional to our comprehension of the intriguing mechanisms by which alterations in cellular NAD metabolism can be linked to changes in gene expression. Thus, NAD capping may represent a novel frontier in unravelling the intricate interplay between cellular metabolism and RNA biology.

### 3.7 DpCoA TagSeq

**Figure 6:**
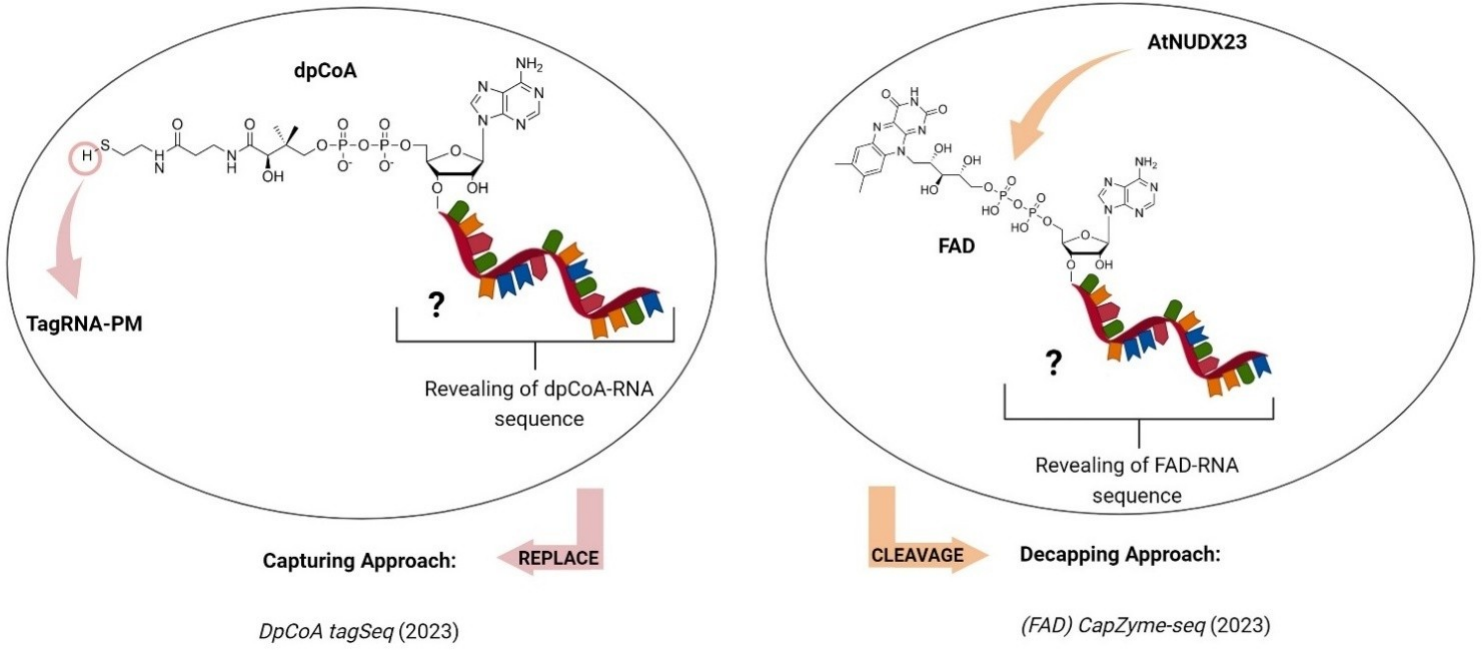
**Left panel**: Sequencing method for the identification of dpCoA‐RNA. In the capturing approach a thiol‐reactive maleimide group (propargyl maleimide, PM) is used to label dpCoA cap with a tag RNA serving as a 5′ barcode. Right panel: Sequencing method for the identification of FAD‐RNA. In the decapping approach, *Arabidopsis thaliana* Nudix pyrophosphohydrolase 23 (AtNUDX23) is used to selectively cleave and target FAD for subsequent analysis. The figure was created using BioRender.com.

Although dpCoA has been recognized as an RNA cap in various bacterial species,[^18^, ^66^] the identities of these RNA remain undisclosed. Recently, a new method called dpCoA tagSeq^[98]^ was introduced. Based on the NAD tagSeq^[35]^ concept, this method involves ligation of a tag RNA to the dpCoA cap using a maleimide‐thiol reaction^[99]^ before performing nanopore direct RNA sequencing (Figure 6, left panel). The 5′ RNA tag, acts as a barcode for dpCoA‐RNAs, reducing the likelihood of sequencing other thiol‐containing RNAs (e. g. cysteinyl‐tRNAcys).^[100]^ The tagSeq procedure was tested by identifying dpCoA‐RNAs in mixed samples containing synthetic model dpCoA‐RNA and real RNA extracts from mouse liver. In an initial attempt, 44 putative dpCoA‐RNAs were identified^[98]^; however, these were later revealed to be false positives due to nanopore sequencing errors.^[101]^ Issues such as chimeric reads merging model RNA spike‐ins with mouse RNAs and incorrect mapping of reads to multiple genes were addressed by implementing measures like eliminating reads containing the tagged model RNA sequence and filtering out reads with a 5′ end clip length ranging from 20–100 nt.^[102]^ This refined analysis significantly reduced the number of tagged RNA reads, resulting in seven potential dpCoA‐RNA candidates.^[102]^ However, additional confirmation of these genes might be necessary to validate the method and to confirm that these transcripts indeed bear dpCoA RNA cap. Here lies the crucial importance for meticulous and thorough bioinformatic analysis, along with implementing a filtering process to exclude any reads containing the spike‐in model sequence. It is also crucial to employ orthogonal approaches to independently confirm any hits obtained by sequencing methods in general. This work is currently the only work focused on the identification of the dp‐CoA‐RNA sequence. Due to its limitations mentioned previously, there is an urgent need for new, reliable techniques to uncover the identity and biological role of dpCoA‐RNAs.

### 3.8 FAD CapZyme‐Seq

Although the role of FAD as a potential NCIN has been known for many years,[^14^, ^15^, ^19^, ^21^] Sherwood et al. were the first to observe FAD covalently attached to the HCV RNA,^[20]^ providing a new example of viral 5′‐metabolite capping and demonstrating a functional role for FAD capping.^[20]^ They adapted the previously developed (NAD) CapZyme‐seq method^[65]^ using the recombinant *Arabidopsis thaliana* Nudix pyrophosphohydrolase 23 (AtNUDX23), which is highly specific for FAD (Figure 6, right panel).^[103]^ To distinguish between 5′‐ppp RNAs and 5′‐FAD RNAs, RNA 5′‐polyphosphatase (Rpp), which specifically cleaves RNA 5′‐ppp ends,^[65]^ was used in parallel. Both the positive strand RNA genome (HCV(+)) and the reverse complementary negative strand (HCV(−)) were notably enriched in the AtNUDX23‐treated libraries.^[20]^ They estimated that approximately 75 % of the 5′ termini of HCV(+) and HCV(−) were FAD‐capped, with the remaining 25 % having a 5′‐p cap.^[20]^ This is the highest frequency observed for any metabolite‐capped RNA across all kingdoms of life.[^31^, ^34^, ^39^, ^60^] LC–MC analysis further validated the sequencing results.

Recently, a method was developed for capturing 5′‐thiamine‐capped RNA by inserting biotin through thiazole ring‐opening nucleophilic substitution combined with CuAAC.^[104]^ However, this type of RNA has only been synthesized *in vitro* and has not yet been identified in the RNA of any organisms. Further studies are required to validate the existence of such a 5′ RNA cap.

## 4 Future Potential Techniques for the Identification of Non‐Canonical Capped RNAs

**Figure 7:**
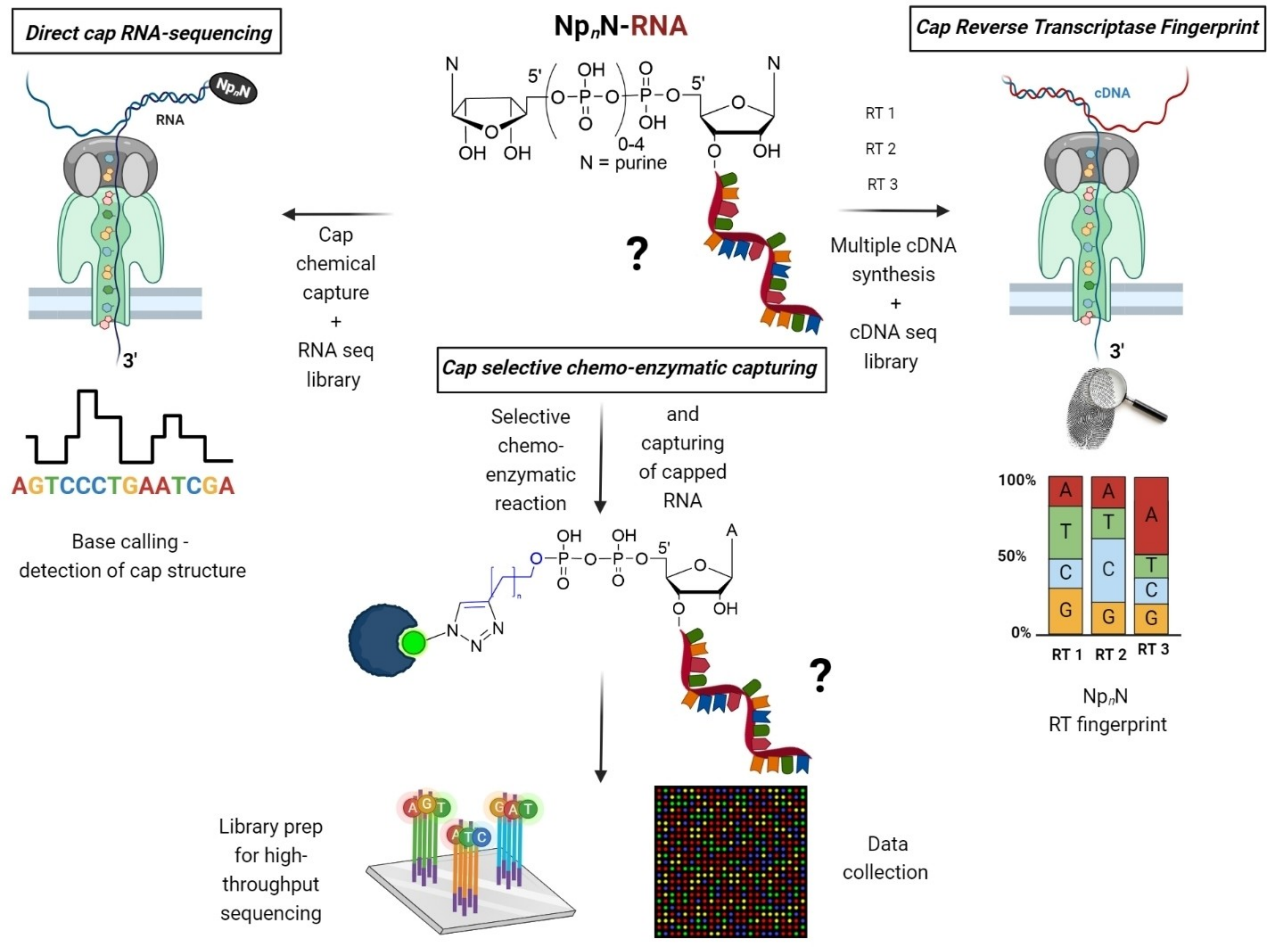
Future potential techniques for the identification of non‐canonical capped RNAs (e. g. Np_*n*_Ns). Direct cap RNA‐sequencing. Cap selective chemo‐enzymatic capturing. Cap Reverse Transcriptase (RT) fingerprint. The figure was created using BioRender.com.

The lack of exclusive sequencing methods to reveal Np_*n*_Ns‐RNAs identity in both bacterial and eukaryotic cells has become a major obstacle in understanding the role of these RNA caps. To address this issue, several techniques are currently being developed. One of the options is the direct nanopore sequencing of RNA bearing NCINs (direct capRNA‐seq) (Figure 7). This technique has the great advantage of sequencing RNA directly and in real time, without the need for amplification. Additionally, nanopore direct sequencing generates longer read lengths than Illumina sequencing, facilitating the sequencing of full‐length RNA transcripts and the identification of complex transcript isoforms. These advantages make nanopore direct RNA sequencing particularly valuable for preserving native RNA features and enabling comprehensive transcriptome analysis. Although the structural similarity between Np_*n*_Ns poses a challenge for their recognition, nanopore technology can differentiate based on the number of phosphates, creating distinct squiggle patterns. The challenging part is the development of a specific chemical capturing technique for Np_*n*_Ns because they contain canonical nucleotides. Thus, methods to capture RNA containing Np_*n*_Ns must be carefully designed and confirmed by an orthogonal approach such as LC–MS analysis of specific transcripts. This approach would significantly advance the study of both canonical and non‐canonical RNA caps across various organisms, offering a comprehensive solution for researchers. One limitation of nanopore technology is that sequencing begins at the 3′ end and ends at the 5′ end of the RNA, meaning the last 20–25 nucleotides, including the cap, might not be precisely read by the sequencer. Therefore, a biochemical approach that will solve this problem in combination with bioinformatic analysis will be crucial for developing machine learning tools to recognize typical squiggle patterns for model RNA caps and applying this method to RNA from various cellular sources.

Another promising method involves the development of a selective chemo‐enzymatic approach using e. g. various NudiX enzymes (Figure 7). Recently, some plant NudiX enzymes have been reported to be selective towards particular non‐canonical RNA caps.^[51]^ NudiX enzymes may potentially accept nucleophiles such as alkynyl alcohols with a clickable group instead of water for the polyphosphate hydrolysis. This may enable the attachment of a clickable group to RNA during selective cap cleavage. Similar to the NAD captureSeq method,^[31]^ this reaction introduces a triple bond that can undergo CuAAC with biotin azide. The resulting RNA, tagged with biotin, can be captured on streptavidin beads and used to prepare standard Illumina RNA‐seq libraries. A reliable negative control, such as the absence of a NudiX enzyme, is crucial for bioinformatic identification of enriched RNAs with Np_*n*_Ns caps and filter out false positives caused by high concentrations of certain RNAs such as rRNA and tRNA. The identification of specific decapping enzymes that selectively cleave particular caps is key to the success of this approach. Further efforts are needed to isolate additional NudiX enzymes from sources like *E. coli*, human, or plant cells to ensure comprehensive coverage of the full range of detected caps.

Reverse transcription of RNA templates that contain internal RNA modifications leads to cDNA synthesis that incorporates information about these modifications, manifesting as misincorporation, arrest, or nucleotide skipping events.^[105]^ The collection of such occurrences across multiple cDNAs generated by various reverse transcriptases (RTs) forms an RT signature characteristic of a particular modification. Machine learning tools have recently been employed to discern internal RNA modifications, such as 6‐methylguanosine (m⁶G) and 6,6‐dimethyladenosine (m₂⁶A), from these RT signatures.[^106^, ^107^] Another encouraging approach could employ the RT fingerprinting method to identify 5′ RNA caps (Figure 7). To evaluate the capabilities of RTs in reading and recognizing Np_*n*_Ns caps, model RNAs bearing different 5′ caps should be tested with a combination of available RTs. Subsequently, cDNA sequencing libraries suitable for either Illumina or ONT are prepared. The distinct behaviour exhibited by various RTs in response to different RNA caps, analysed through machine learning, validates the method's potential for application to real RNA from bacterial or eukaryotic cells. Ensuring high reproducibility of RT behaviour and the production of high‐quality cDNA‐seq libraries are instrumental for establishing a reliable method applicable to real RNA samples. However, owing to the prevalence of the canonical cap in mRNA of eukaryotic cells, the applicability of this method to the entire transcriptome is constrained. To address this limitation, pre‐enrichment of the cellular RNA pool becomes necessary, either by eliminating m⁷G‐capped RNAs or by enriching specific non‐canonical capped RNAs using decapping enzymes.

## 5 Summary and Outlook

The field of RNA biology is currently experiencing an extraordinary period of rapid advancement, with a significant influx of new information. The challenge lies in comprehensively understanding the integrated mechanisms governing gene expression. We now need to consider the complexities of chromatin modifications and epigenetics, as well as the regulation of transcription and both co‐transcriptional and post‐transcriptional processes. Non‐canonical RNA capping adds a layer of epitranscriptomic regulation by modulating RNA stability and translation efficiency to 5′‐canonical mRNA capping in eukaryotes. This implies that not only in eukaryotes, but also in organisms evolutionarily distant and originally considered “simple”, there exists a system of 5′ non‐canonical capping whose role remains unclear. To fully understand the impact of non‐canonical capping on RNA fate is essential to study the mechanisms that determine the distribution of non‐canonical RNA caps across different subsets of transcripts. The significance of developing specialized techniques for identifying non‐canonically capped RNAs is directly tied to the importance of understanding these RNAs, including their structure, function and cellular localization. Such techniques can shed light on their functions and offer new insights into cellular processes and molecular mechanisms. Moreover, in a broader framework, dysregulation of canonical mRNA capping has been associated with various diseases, including cancer[^108^, ^109^, ^110^] and viral infections.[^111^, ^112^, ^113^] Non‐canonical caps may be indicative of abnormal RNA processing pathways or viral hijacking of host cellular machinery. Therefore, detecting non‐canonical capped RNAs could aid in diagnosing diseases and developing therapeutic interventions. Finally, RNA capping holds significant importance in diverse biotechnological endeavours, including mRNA‐based therapeutics and RNA vaccines. The presence of non‐canonical caps has the potential to influence the stability, translation efficacy and immunogenicity of synthetic RNAs. Therefore, the development of methodologies for identifying and analysing non‐canonical caps is also crucial for enhancing the formulation and effectiveness of RNA‐based therapeutics. We believe that this review conveys the urgency of developing novel non‐canonical RNA cap‐specific enrichment and sequencing protocols for identifying these RNAs. Such advancements are pivotal for broadening our comprehension of RNA biology, uncovering new disease biomarkers and advancing potential therapeutic interventions targeting 5′ end conjugates.

## Conflict of Interests

The authors declare no conflict of interest.

## Biographical Information

*Flaminia Mancini, M.Sc., is a geneticist and molecular biologist specializing in RNA biology. She received her Bachelor's degree in Biotechnology from the University of Tuscia, Italy, where her fascination with genetic mechanisms was first ignited. She continued her studies at the University of Rome “La Sapienza,” earning a Master's degree in Genetics and Molecular Biology, with a focus on RNA biology and gene expression regulation. Her thesis investigated the control of genome stability in Drosophila embryos. Currently, as a Ph.D. student at IOCB, Prague, Flaminia focuses on developing sequencing techniques to study RNA modifications, particularly 5’ non‐canonical capping and its biological role*.

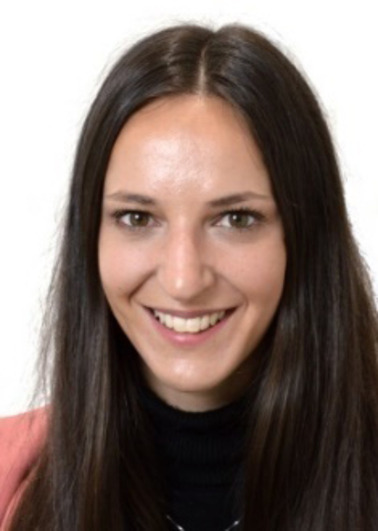

## Biographical Information

*Hana Cahova received her PhD at IOCB Prague and UCT Prague under supervision of Prof. Michal Hocek in 2010. Afterwards, she conducted her postdoctoral stay with Prof. Andres Jäschke at Heidelberg University. As a Humboldt fellow, she studied NAD‐RNA and DNA photoswitches. In 2016, she established her independent junior research group at IOCB Prague. With her team, she focuses on RNA modifications in model systems such as viruses or bacteria. In 2022, she received ERC Starting grant to study dinucleoside polyphosphate RNA capping and its role*.